# Novel humanized CD19-CAR-T (Now talicabtagene autoleucel, Tali-cel™) cells in relapsed/ refractory pediatric B-acute lymphoblastic leukemia- an open-label single-arm phase-I/Ib study

**DOI:** 10.1038/s41408-025-01279-9

**Published:** 2025-04-24

**Authors:** Gaurav Narula, Swaminathan Keerthivasagam, Hasmukh Jain, Sachin Punatar, Akanksha Chichra, Chetan Dhamne, Prashant Tembhare, Papagudi Ganesan Subramanian, Nikhil Patkar, Minal Poojary, Anant Gokarn, Sumeet Mirgh, Nishant Jindal, Albeena Nisar, Deepali Pandit, Khushali Pandit, Alka Dwivedi, Atharva Karulkar, Ankesh Kumar Jaiswal, Aalia Khan, Shreshtha Shah, Afrin Rafiq, Moumita Basu, Juber Pendhari, Sweety Asija, Ambalika Chowdury, Ankit Banik, Nirmalya Roy Moulik, Shyam Srinivasan, Shilpushp Bhosle, Sumathi Hiregoudar, Shashank Ojha, Lingaraj Nayak, Jayshree Thorat, Bhausaheb Bagal, Manju Sengar, Navin Khattry, Shripad Banavali, Steven Highfill, Nirali N. Shah, Rahul Purwar

**Affiliations:** 1 Department of Pediatric Oncology, Tata Memorial Centre, Homi Bhabha National Institute, Mumbai, India; 2 Department of Medical Oncology, Jawaharlal Institute of Postgraduate Medical Education and Research, Puducherry, India; 3 Department of Medical Oncology, Tata Memorial Centre, Homi Bhabha National Institute, Mumbai, India; 4 Bone marrow transplant unit, Department of Medical Oncology, Tata Memorial Centre, Advanced Centre for Treatment, Research and Education in Cancer (ACTREC), Homi Bhabha National Institute, Mumbai, India; 5 Department of Hematopathology, Tata Memorial Centre, Advanced Centre for Treatment, Research and Education in Cancer (ACTREC), Homi Bhabha National Institute, Navi Mumbai, India; 6 Department of Transfusion Medicine, Tata Memorial Centre, Advanced Centre for Treatment, Research and Education in Cancer (ACTREC), Homi Bhabha National Institute, Navi Mumbai, India; 7 Scientific Officer (D), CAR-T and Cell Therapy Centre, ACTREC, Tata Memorial Centre, Mumbai, India; 8 CAR-T and Cell Therapy Centre, ACTREC, Tata Memorial Centre, Kharghar, India; 9 Department of Biosciences & Bioengineering, Indian Institute of Technology Bombay, Mumbai, India; 10 Pediatric Oncology Branch, Center for Cancer Research, National Cancer Institute, National Institutes of Health, Bethesda, MD USA; 11 Immunoadoptive Cell Therapy Private Limited (ImmunoACT), Mumbai, India; 12 Department of Critical Care and Anaesthesiology, Tata Memorial Center, Homi Bhabha National Institute, Mumbai, India; 13 Director Academics and Professor Medical Oncology, Tata Memorial Center, Homi Bhabha National Institute, Mumbai, India; 14 Center for Cellular Engineering, National Cancer Institute, National Institutes of Health, Bethesda, MD USA

**Keywords:** Acute lymphocytic leukaemia, Targeted therapies

## Abstract

**Abstract:**

Chimeric Antigen Receptor-T (CAR-T) cell therapy is effective for relapsed/refractory B-acute lymphoblastic leukemia (r/r B-ALL) but is not universally available. We developed a novel humanized CD19-directed CAR-T (HCAR19) approved for Phase 1/1b/2 trials. Patients aged 3–25 years were enrolled with r/r B-ALL and ineligible for allogeneic stem cell transplant. Lymphodepletion utilized standard-dose fludarabine and cyclophosphamide. A 3 + 3 design testing 3 dose-ranges was used to determine Phase-2 Dose (P2D): Dose-A, 1 × 10^6^ HCAR19 cells/kg, Dose-B, 3–5 × 10^6^/kg, and Dose-C, 10–15 × 10^6^/kg. Primary endpoint was overall response rate (ORR) at day-30 on bone-marrow flow-cytometry. From May-2021 to September-2023 12 patients [median age-14 (range: 5–24) years] were enrolled with median bone marrow blasts 19.5% at screening. Cytokine release syndrome occurred in 10 (83%) patients, predominantly Grades 1–2, and Grade-2 immune-cell associated neurotoxicity (ICANS) in 1. All patients had Grade-3 cytopenia. ORR was 91.7% (11/12), complete response (CR) in 8 (66.7%) and partial response in 3 (25%). Seven of 8 CRs were at Dose-levels B and C, all of which were sustained till 12 months follow-up. Patients who received dose levels below 3 × 10^6^/kg, or did not achieve CR, had early loss of response or rapid progression. HCAR19 demonstrated safety, manageable toxicity, and durable remissions. and P2D was determined as 5–10 × 10^6^ HCAR19-cells/kg.

**Clinical trial registration:**

The study is registered in the Clinical Trials Registry- India (CTRI/2021/05/033348 and CTRI/2023/03/050689).

## Introduction

B-Acute lymphoblastic leukemia (B-ALL) in children is highly curable with significant strides in outcomes made each decade. Contemporary risk-stratified and response-adapted pediatric protocols for B-ALL have resulted in survival rates exceeding 90% in developed countries [1, 2]. Over time, the use of “pediatric-like” protocols has also improved prognosis in adolescents and young adults (AYAs) [3]. However, the outcomes for pediatric and AYA patients with relapsed/refractory (r/r) B-ALL remain poor [4–6]. Subsets of these patients such as those with very early relapses, relapses after allogeneic stem cell transplants (Allo-SCT), and minimal residual disease (MRD)-positivity post-reinduction do not fare well with the available therapies. The introduction of immunotherapies, such as bi-specific T cell engagers (BiTEs) and antibody-drug conjugates (ADCs), has improved outcomes in these groups but remains largely inaccessible in low- and middle-income countries (LMICs) [7–10].

Chimeric antigen receptor (CAR) T-cell therapy, a type of adoptive cell immunotherapy, has shown dramatic responses in the treatment of r/r B-ALL leading to the approval of the first CAR T-cell product, tisagenlecleucel (Kymriah®, Novartis), by the US Food and Drug Administration (FDA) in 2017 [11, 12]. Subsequent approvals of CAR T cells directed against other B-cell malignancies and multiple myeloma have shown similar results across various products over time in trials, and through real-world experience [13–18]. However, a common challenge with these therapies has been their prohibitive cost, making them inaccessible to many LMICs as well as some developed countries. Foreseeing the continued lack of access to these therapies in India, we set about addressing this with a collaborative project between the Indian Institute of Technology Bombay (IIT B) and Tata Memorial Centre (TMC) on CAR T-cells initiated in 2015, by developing indigenously, a novel humanized CD19-directed CAR T product- HCAR19. We collaborated with investigators at the National Cancer Institute (NCI), Bethesda, MD, USA, to train and manufacture indigenously engineered, humanized HCAR-19. These efforts resulted in robust preclinical data and demonstrated clinical-grade manufacturing capabilities [19]. We then obtained regulatory approval from central regulatory agencies in India to initiate a pilot phase 1 trial of HCAR-19 in pediatric, adolescent, and young adult patients with r/r B-ALL (PAYALL). The PAYALL trial was followed by a roll-on Phase 1b/2 trial. Here, we report the per-protocol planned pooled analysis of the pilot Phase 1 and Phase 1b trials used to determine the Phase 2 dose (P2D) in PAYALL patients with r/r B-ALL.

## Methods

### Trial design

Being the first-in-country trial of a cell and gene therapy in a pediatric population, the initial regulatory approval faced many challenges due to evolving guidelines for such products. The study was approved by regulatory oversight bodies, including the Institutional Ethics Committee (IEC), Institutional Biosafety Committee (IBSC), the Review Committee on Genetic Manipulation (RCGM) of the Government of India, and the Central Drugs Standard Control Organisation (CDSCO). The initial approval was for a pilot Phase I trial in only six patients 3–25 years of age to test the feasibility and safety of the indigenously engineered novel HCAR-19, incorporating a 4–1BB co-stimulatory domain, (now talicabtagene autoleucel, Tali-cel™) in r/r B-ALL pediatric patients, manufactured at IIT B as previously described [19]. The trial tested one dose level of 1 × 10^6^ CAR T-cells/kg body weight, amended after the first three patients to a second dose level of 3–5 × 10^6^ CAR T-cells/kg body weight. The trial was registered with the Clinical Trial Registry of India (CTRI) (CTRI/2021/05/033348). Subsequently, a Phase 1b/2 trial was initiated (CTRI/2023/03/050689), where a third dose level of 10–15 × 10^6^ CAR T-cells/kg body weight was proposed to determine the optimal phase 2 dose (P2D), which would then be rolled into an extension Phase 2 cohort. Data across all 3 dose levels from both trials was eligible for P2D determination. The clinical trials were conducted at TMC, while manufacturing occurred at IIT-B.

### Ethics approval and consent

The prospective study was approved by the institutional review boards and ethical committee clearance was obtained (ECR/149/Inst/MH/2023 and IITB-IEC/2018/023). All clinical investigations were conducted according to the principles of the Declaration of Helsinki. Informed consent was obtained from all study participants or their legal guardians.

### Inclusion and exclusion criteria

**Fig. 1:**
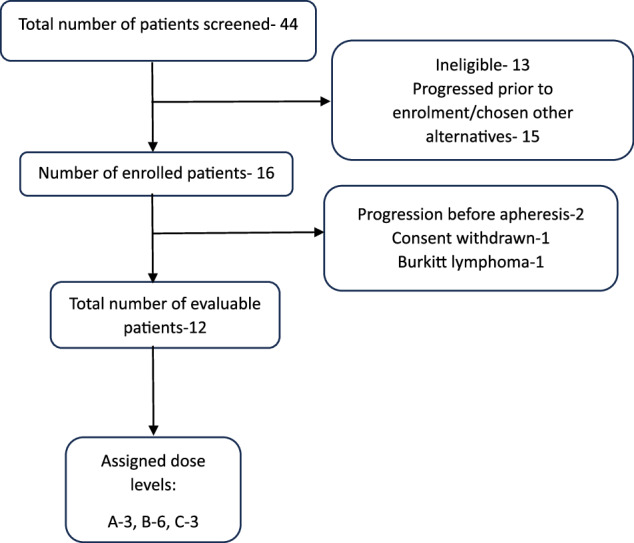
Study course for participants from the time of enrollment to treatment. Dose levels- Dose-A: 1 × 10^6^/kg, Dose-B: 3–5 × 10^6^/kg, Dose-C:10–15 × 10^6^/ kg body weight.

Patients with r/r B-ALL aged 3–25 years who were ineligible for allogeneic stem cell transplant (Allo-SCT) or unable to proceed with an indicated SCT for any other reason, having no prior history of CD19-directed therapies were included if CD19 expression exceeded 99% in the blast population in the absence of other detectable clones. The analysis period for both studies covered patients enrolled between May 2021 and September 2023, with a subsequent one-year follow-up post-infusion of HCAR-19 cells in the final patient, extending through September 2024. Patients were excluded if they had isolated extramedullary relapse, active CNS disease, major comorbidities including organ dysfunction, active chronic infections, graft-versus-host disease, cancer predisposition or genetic syndromes, Allo-SCT within six months before screening-amended later to 3 months post-Allo-SCT, were positive on urine pregnancy tests, pregnant or lactating, or were receiving an investigational medicinal product within 30 days of screening. Other CD19-expressing r/r malignancies in the defined age group, such as Burkitt lymphoma, diffuse large B-cell lymphoma, primary mediastinal B-cell lymphoma, and primary CNS lymphoma, were also included in the Phase 1B trial on a compassionate basis. However, this analysis is restricted to B-ALL patients. A solitary patient of Burkitt Lymphoma enrolled is described in Supplementary Table 1. The study course of participants is shown in Fig. 1.

### Apheresis, manufacture, and infusion of CAR

Patients with r/r B-ALL were screened for apheresis and considered eligible if CD3 count was more than 150/μl. Lymphocyte-collection protocol on FreseniusKabiCom.Tec™ was employed. The collected sample was transported to the CAR-T manufacturing site at IIT-B. In the interim, patients continued physician-determined bridging chemotherapy to decrease the disease burden, prevent disease progression, or to maintain a sustained remission, if achieved, before the infusion of CAR-T cells. Disease-directed therapy was discontinued 7 days before the lymphodepletion chemotherapy.

Patients received lymphodepletion with fludarabine 30 mg/m^2^ daily for 3 days (days -4 to -2) and cyclophosphamide 500 mg/m² for 2 days (days -3 and -2), before CAR-T cell infusion (Day 0). After passing the release criteria, cryopreserved CAR-T cells were transported to the patient site in a cryo-transporter on the day of infusion. Thawing was performed at the bedside, beginning with a separate tube frozen alongside the product for a viability check. The dose was then recalculated based on the revised viability to ensure accurate dosing followed by the thawing of the product itself. The first three patients received HCAR-19 at a dose of 1 × 10^6^/kg (Dose-A). If no dose-limiting toxicities were observed, two dose-escalation levels were planned for subsequent patients: Dose-B (3–5 × 10^6^/kg) and Dose-C (10–15 × 10^6^/kg body weight).

### Endpoints

The primary efficacy endpoint was the overall response rate (ORR) on day 30 bone marrow evaluation, using multiparametric color flow cytometry (MFC) to assess MRD. Complete response (CR) was defined as MRD < 0.01%. Morphological remission (blasts <5%) with MRD positivity (≥0.01%) was considered a partial response (PR), subject to at least a 1-log reduction in pre-infusion blasts in bone marrow assessment by MFC. The secondary endpoints were to analyze the persistence and dynamics of CAR-T cells, study CAR-T cell toxicities in the population, examine cytokine levels, and conduct follow-up bone marrow evaluations at 2, 3, and 6-months post-infusion. Patients were followed up for 12 months, after which they were considered off-trial but remained under extended follow-up for a planned 5 years, and potentially longer, as convenient for the patient. Cytokine release syndrome (CRS) and neurotoxicity were graded according to the guidelines published by the American Society for Transplantation and Cellular Therapy (ASTCT) [20].

### Antibiotic and antifungal prophylaxis

At our institute, patients received voriconazole, acyclovir and cotrimoxazole for antifungal, antiviral and anti-Pneumocystis jirovecii prophylaxis, as per the prevailing practices in the Stem Cell Transplant unit of our center based on observed patterns of infections, while antibiotic prophylaxis is not routinely used.

### Statistical analysis

A standard 3 + 3 dose-escalation design was used for assessing safety across different CAR-T cell doses. A planned sample size of a minimum of 22 patients was based on Simon’s two-stage design, ensuring power (0.9) to detect a significant response rate (≥50%) compared to a null hypothesis of 20%. In the first 10 patients, 2 or fewer responses would determine futility, else the study would progress. Statistical significance was defined as *p* < 0.05. The primary endpoint ORR was determined as the proportion of patients achieving CR or PR at the 30-day bone marrow evaluation, with 95% confidence intervals calculated using the binomial exact method. This study reports on safety end-points and Phase-2 Dose (P2D) determination.

## Results

### Patient characteristics

The demographic details of the 12 patients with B-ALL who received HCAR-19 are summarized in Table 1. The median age of the cohort was 14 years (range: 5–24) with a male-to-female ratio of 2:1. The median percentage of bone marrow blasts at the time of screening was 19.5% (range: 0.1–88%). Most patients were heavily pretreated, having received at least two lines of therapy before CAR-T cell infusion (median: 2, range: 2–6). Three patients had undergone Allo-SCT, two of whom had received transplants twice. Two-thirds of the patients (8 out of 12) exhibited high-risk cytogenetic and/or molecular abnormalities, the most common being t(9;22) observed in four patients.

**Table 1:** Demographics of patients enrolled in the study cohort.

Patient (P)	Sex	Age at CAR infusion (years)	Disease status at the time of CAR-T infusion	High-risk cytogenetic and molecular aberrations	Number of treatment lines before HCAR-19	BM blasts at the time of enrollment (%)	Pre-infusion MRD (%)
P1	M	18	Late relapse (MRD+ve post-reinduction)	IKZF1del/PAX5-JAK2 fusion	2	1	0.09
P2	F	16	Refractory	–	3	3	28.2
P3	F	11	Very early relapse	t(9,22)/T315I variant	4	72	37.1
P4	M	8	Early relapse (MRD+ve post-reinduction)	–	2	61	0.15
P5	M	24	2^nd^ relapse (post Allo-SCT)	t(9,22)	5	51	51
P6	F	8	Early relapse (MRD+ve post-reinduction)	IKZF1	3	88	0
P7	M	12	Refractory	IKZF1del/PAX5-JAK2 fusion	3	2	0
P8	M	16.5	Refractory	TP53 mutation	3	5	42
P9	F	19	Refractory (ph +ve)	t(9,22)	2	34	0
P10	M	8	Early relapse (MRD+ve post-reinduction)	–	2	0	0
P11	M	16	3^rd^ relapse-refractory (Post 2^nd^ Allo-SCT)	t(9,22)	5	4	4
P12	M	5	Relapse-Refractory (Post 2^nd^ Allo-SCT)	–	6	0	0

Timing of relapse- Very early <18 months, early- 18–36 months, late- >36 months.

*BM* bone marrow, *MRD* minimal residual disease, *Allo-SCT* Allogenic stem cell transplant.

### CAR-T cell infusion

CAR-T cells were successfully manufactured for all 12 patients who underwent apheresis (100%). The median time from enrollment to infusion was 44 days (range: 22–137). The median vein-to-vein time (from apheresis to CAR-T cell infusion) was 27.5 days (range: 18–133). Two patients experienced longer delays: one due to disease progression that required salvage chemotherapy and another due to sepsis followed by a COVID-19 infection. Bridging chemotherapy was used at the discretion of the treating oncologists, typically involving less myelotoxic agents such as vincristine, pegylated l-asparaginase, low to medium doses of cytarabine, cyclophosphamide and etoposide, or oral metronomic therapy with 6-mercaptopurine, etoposide, and/or steroids. Three dose levels were assigned: Dose A (1 × 10^6^/kg) for 3 patients, Dose B (3–5 × 10^6^/kg) for 6 patients, and Dose C (10–15 × 10^6^/kg) for 3 patients. The median number of viable CAR-T cells was 229.5 million cells (range: 42–500), and the percentage of transduced cells was 33.5% (range: 15–65). The maximum dose received by patients was 10 × 10^6^/kg CAR-T cells.

### Safety of CAR infusion

No dose-limiting toxicities were observed. The observed toxicities and their grading at different dose levels are tabulated in Table 2. The most common toxicity observed was CRS, noted in 10 out of 12 patients (83%); peak CRS was Grade 1 in 7 patients (58%), Grade 2 in 1 patient (8%), and Grade 3 in 2 patients (17%). Tocilizumab rescue was required for 5 patients (41.7%), as it was used in all 3 Grade 2/3 CRS events and also in 2 Grade 1 CRS events. One patient required vasopressors while managing CRS. One patient experienced a peak CRS of Grade 3 on day 4; this patient also had a prior severe COVID-19 infection during the apheresis-to-infusion period and a high tumor burden before CAR-T cell infusion. The patient had significant capillary leak syndrome during the COVID-19 episode, which was managed with Paxlovid, steroids, and an antibody cocktail, allowing for recovery. However, due to significant weight gain over pre-existing obesity, the manufactured CAR T-cell dose fell short of the targeted 3 × 10^6^ cells/kg. Baseline IL-6 levels prior to CAR T infusion were 461 pg/ml, with peak levels on day 3 reaching 4312 pg/ml. Additionally, the same patient experienced Grade 2 ICANS noted from day 4 onward, which was managed with intravenous steroids and resolved by week 3 post-infusion. However, this patient had a recurrence of limb weakness in week 6 post-infusion that was presumptively treated for ICANS recurrence with systemic steroids, intrathecal hydrocortisone, Anakinra, and IV immunoglobulins. Although there was a partial response to the treatment, subsequent investigations revealed relapse. The patient had progressive disease thereafter and died from ALL progression. This was the solitary case of neurotoxicity observed in the trial. All patients (100%) experienced Grade 3/4 cytopenia in the immediate post-CAR T infusion phase. Cytopenia resolved by day 30 in 9 out of 12 patients. Those with delayed recovery had either a partial response or no response at the day 30 evaluation. Grade 3 febrile neutropenia occurred in 10 patients (83.3%), with most cases occurring in the pre-infusion period. Eight patients (66.7%) who achieved either CR or PR developed B-cell aplasia (IgG <450 mg/dl) and required monthly intravenous immunoglobulin (IVIg).

**Table 2:** Adverse events of special interest and hematological toxicities.

Toxicities, *n* (%)	All patients (*n* = 12)	Toxicities, *n* (%)	All patients (*n* = 12)
**Cytokine release syndrome**		**3. Febrile neutropenia**	
Grade III	2 (17%)	Grade III/IV	0 (0%)
Grade II	1 (8%)	Grade II	10 (83%)
All Grades	10 (83.3%)	All Grades	10 (83%)
**ICANS**		**4. Anemia**	
Grade III/IV	0(0%)	Grade III/IV	07 (58%)
Grade II	1 (8%)	Grade II	03 (25%)
All Grades	1(8%)	All Grades	12 (100%)
**Hematological toxicities**		**5. Platelet count decreased**	
**1. Cytopenia**		Grade III/IV	06 (50%)
Grade III/IV	12 (100%)	All Grades	10 (83%)
All Grades	12 (100%)	**6. Leukopenia**	
**2. Neutropenia**		Grade III/IV	09 (75%)
Grade III/IV	12 (100%)	All Grades	12 (100%)

*ICANS* immune effector cell-associated neurotoxicity syndrome.

In the extended follow-up period (>3 months), one patient developed pneumonia requiring high-flow nasal cannula in the intensive care unit and achieved complete recovery. The probable cause was influenza virus identified in upper respiratory tract secretions by the Biofire™ assay. Another patient developed abdominal bloating and reduced appetite at month-6 post-infusion. Investigations revealed ascites, abdominal lymph nodes, and hepatic and splenic abscesses, which were confirmed due to mycobacterial tuberculosis. She was managed on anti-tubercular treatment (ATT), to which she responded well. However, at month 8 post-infusion, she was admitted with a severe episode of enterocolitis due to C. difficile, probably secondary to ATT. She recovered from this and has remained well in sustained remission until the last follow-up.

### Clinical response and outcome

**Fig. 2:**
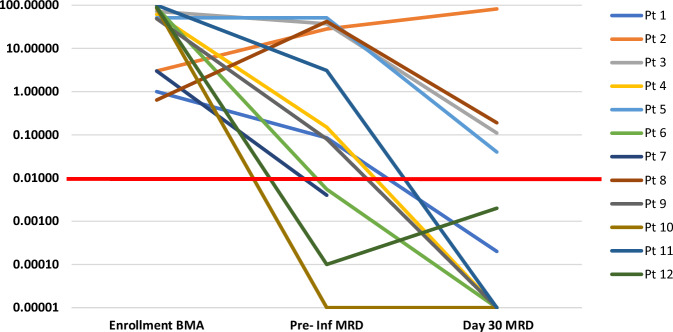
Log-reduction of disease in the bone marrow by flow cytometry on day 30 after HCAR-19 infusion for each patient. BMA bone marrow aspirate, pre inf MRD pre-infusion minimal residual disease.

Primary end-point: Response rates at each time point and different dose levels are tabulated in Table 3. On day 30 bone marrow evaluation, ORR for the entire cohort was observed to be 91.7% (11 out of 12 patients), of which 8 (66.7%) achieved a CR and 3 (25%) had a PR, while one had no response (NR). Across all eight patients who received either dose B or C (i.e., ≥3 × 10^6^ cells/kg), ORR was 100%. Among these, one patient with PR experienced disease progression by month 2, while all the remaining 7 patients had sustained remissions at months 3 (7/7), 6 (7/7), and 12 (7/7) post-CAR T infusion. Log-reduction of disease in the bone marrow of each patient after the infusion of HCAR-19 is shown in Fig. 2.

**Table 3:** Response rates at different time points post-HCAR19 infusion.

Patients	Response	Day 30	Day 60	Day 90	Day 180
Whole cohort: *n* = 12	CR	8 (67)	8 (67)	7 (58)	5 evaluable patients- All in sustained remission
	PR	3 (25)	0 (0)	1 (8)	
	NR	1 (8)	4 (33)	–	
	ORR	11 (92)	8 (67)	8 (67)	
Dose level A^a^ (<3 × 10^6^/Kg): *n* = 4	ORR	3 (75%)	1 (25%)	0	Not evaluable
Dose level B and C (≥3 × 10^6^/Kg): *n* = 8	ORR	8 (100%)	7 (88%)	7 (88%)	5 (63%)

An MRD cutoff of <0.01% is defined as negative, while ≥0.01% is considered positive.

*ORR* Overall response rate, *CR* complete remission (minimal residual disease-negative), *PR* partial remission (morphological remission, minimal residual disease-positive).

^a^A patient who received only 2.13 × 10^6^ HCAR-19 cells/kg was assigned to dose level A for analysis.

Of the 8 patients who achieved CR, 3 received further therapy. This included 2 patients who lost B-cell aplasia within 6 months of CAR-T infusion, one of whom underwent Allo-SCT, while the other is on oral maintenance chemotherapy. One additional patient who received dose A lost his MRD response at 3 months and underwent further therapy and Allo-SCT. At the 3-month follow-up, sustained MRD negativity was noted in 7 out of 8 patients (87.5%) who achieved CR on day 30. All patients with PR had a very short duration of response and relapsed and progressed rapidly thereafter. One of them had a CD-19 negative relapse.

**Fig. 3:**
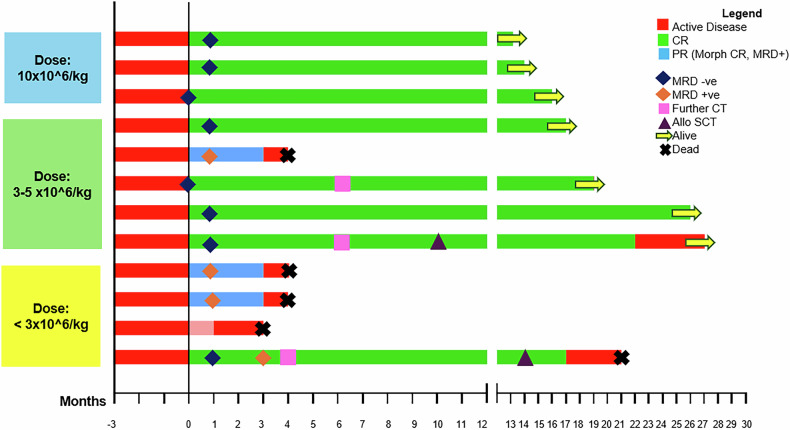
Swimmer’s Plot predicting responses and follow-up across varied dose levels of HCAR-19 therapy. CR complete remission (minimal residual disease-negative), PR partial remission (morphological remission, minimal residual disease-positive), CT chemotherapy, Allo SCT allogenic stem cell transplant. An MRD cutoff of <0.01% is defined as negative, while ≥0.01% is considered positive.

In extended follow-up, 8 patients were alive at 1 year or beyond from the entire cohort. Two of these had late relapses 21 and 24 months post CAR T infusion after subsequent therapies and allogeneic stem-cell transplants, of which one is alive on 5th-line therapy at the time of analysis. The remaining 6 are in sustained remissions beyond 1-year follow-up. Swimmer’s plot of responses and follow-up across varied dose levels of HCAR-19 therapy are shown in Fig. 3.

### In-vivo dynamics and their correlation of clinical outcomes and responses

**Fig. 4:**
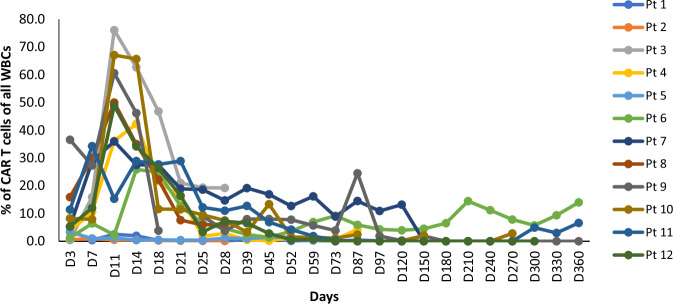
Expansion and persistence of HCAR-19 in each patient at different time-points. WBC white blood cells.

**Fig. 5:**
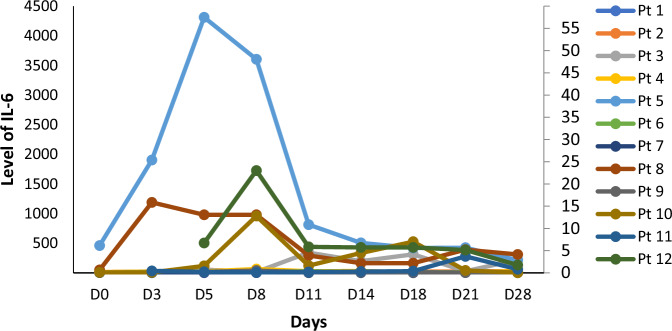
IL-6 levels monitored at different time points for each patient.

HCAR-19 was detected in the peripheral blood through MFC in all patients. The expansion and persistence of HCAR-19 in each patient at different time points is shown in Fig. 4. The median time to peak expansion of CAR-T cells was 12.5 days (range: 7–25). No relationship between the dose and expansion of HCAR19 was observed. The peak expansion of HCAR19 correlated well with IL-6 levels, however there was no correlation with the other cytokines (Fig. 5). Overall, cytokine production was low. Peak IL-6 levels were 23.31 pg/ml (range: 4.6–4312 pg/ml) at a median duration of 8 days from infusion (range: 3–11). Similar patterns were seen for IFN-γ and IL-8 with Day-8 median levels 5.56 pg/ml (range: 0–65.3 pg/ml), and 35.7 pg/ml (range: 1.61–7.02 pg/ml) respectively. Levels of other cytokines like IL-10, IL-2, MIP1$\alpha$, and GM-CSF were similar or even lower (Supplementary Fig. 1). A solitary patient with pre-infusion high IL-6 levels from prior sepsis and COVID-19 accounted for the high upper end of the range skewed from the remaining data for all cytokines measured.

## Discussion

Treating r/r ALL in low- and middle-income countries remains challenging due to financial constraints, long wait times for Allo-SCT, and limited access to advanced therapies like CAR-T cells and blinatumomab [21–24]. The global disparity in access to CAR-T cell therapy stems significantly from the high costs associated with manufacturing CAR-T cells and the expertise required to manage the novel toxicities that arise in patients following CAR-T cell infusion. This led us to develop and bring to trial a novel indigenously designed CD19-directed CAR T product.

Standard doses of fludarabine and cyclophosphamide were used for lymphodepletion therapy, similar to other studies, and were well tolerated [25–27]. While the efficacy of CAR-T cells has been recently described in patients with CNS disease [28], active CNS disease was excluded from our trial and remains unassessed. However, we did observe the persistence of HCAR19 in the CSF at different time points in our patients.

Toxicities related to CAR-T cell infusion remained manageable even at higher dose levels. Our pre-clinical studies also had demonstrated a favorable toxicity profile with decreased production of cytokines (TNF-ɑ and IFN-γ) [19]. Among the cohort of 12 patients, only 2 individuals (17%) experienced grade-3 CRS. Both of these patients had high disease burden before HCAR19 infusion and did not correlate with dose-level. The incidence of severe CRS (grade 3) was lower at all dose levels compared to rates in other products, which ranged from approximately 23% to 46% [11, 12, 29, 30]. Although all patients in our cohort experienced grade 3/4 cytopenia in the immediate post-infusion phase, none of them required growth factor support or thrombopoietin receptor agonizts. Additionally, none of the patients required intensive care unit (ICU) support within the first 30 days following HCAR19 infusion. Only one patient was initially diagnosed and treated for ICANS but was later found to have CNS relapse upon symptom recurrence. The initial six patients were managed in the bone marrow transplant unit with intensive monitoring until the day 30 response assessment. However, as we gained experience and the toxicities proved manageable and reversible, subsequent patients were kept as in-patient only for the first two weeks. Late toxicities were infrequent and comparable to other reports [31, 32]. One patient notably had sustained lymphopenia during the follow-up period, and had abdominal tuberculosis, which by the known natural history of mycobacterial disease was likely acquired well before CAR-T treatment and possibly unmasked post infusion.

All dose levels produced an overall response rate of around 91% and exhibited robust expansion dynamics at the day 28 evaluation similar to the profiles of other products with 4–1BB co-stimulatory domains. However, meaningful responses of MRD-defined complete response (CR) of 87.5% that were durable beyond 3 months and were seen at dose levels ≥3 × 10^6^/kg. Patients with high-burden disease had less deep and less durable remissions; however, most (75%) of these patients received doses <3 × 10^6^/kg. Our data support lowering disease burden before infusion as a desirable objective, in line with current consensus [27, 33]. Among the 8 patients who achieved CR, a child with the longest follow-up did not undergo any alternative therapy including transplant after HCAR19 infusion and is well 2 years post-HCAR19 infusion, whereas 2 patients underwent Allo-SCT due to various indications, as mentioned earlier. Moreover, it’s noteworthy that two patients who experienced relapse following two prior Allo-SCTs have sustained remission for over 1 year at the last follow-up following an HCAR19 dose of 10 × 10^6^/kg. The need for consolidative HSCT in our patients was determined by the loss of MRD response or loss of B-cell aplasia defined by any reappearance of B cells, which is now recognized as a risk factor for impending relapse [34]. However, not all patients were able to undergo Allo-SCT due to various factors including financial constraints, long waiting lists at the public sector hospitals or reluctance to proceed with second transplant. Oral maintenance therapy is ongoing for one patient in our cohort who experienced early loss of B-cell aplasia (<6 months). This strategy has shown reasonably good outcomes and quality of life [34]. Around 7–25% of patients tend to have CD19-negative relapse; however, in our study, one child (8.3%) had a CD19-negative relapse, which was detected at the end of month 1, indicating the possibility of a pre-existing clone that may have been missed during screening, though a thorough review of the pre-infusion population on flow cytometry did not reveal any such population [35].

The manufacture of CAR-T cells was successful in all patients, demonstrating the capability of a small Good Manufacturing Practice (GMP) facility in an academic institute. Preliminary efficacy data from our phase-I study is promising, with minimal toxicities comparable to CAR-T cell therapies from developed countries [12, 26, 27]. However, a key distinction is cost—our CAR-T cell therapy, including logistics, lentiviral vector expenses, and quality control, is estimated at $30,000 per infusion, compared to $373,000 to $475,000 per infusion in developed countries [36]. This significant cost reduction offers hope for broader accessibility of CAR-T cell therapy in LMICs [37].

For determining the Phase-2 Dose (P2D), we observed robust efficacy at 10 × 10^6^/kg dose-level without dose-limiting toxicities and did not need to test the higher levels of the dose range that went up to 15 × 10^6^/kg, thereby determining the ceiling dose of 10 × 10^6^/kg CAR-T cells. While efficacy was seen in the 3–5 × 10^6^/kg dose too, the solitary partial response and 2 cases of non-durable B-cell aplasia in the first 6 months combined with better results in those who received closer to the upper level of the range led us to propose the lower limit dose of 5 × 10^6^/kg.

In conclusion, this Phase I/IB study conducted at a single center using cost-effective indigenous HCAR-19 therapy demonstrated safety, a manageable toxicity profile, and promising durable remission rates in heavily pretreated r/r B-ALL patients. A recommended dosing range of 5–10 × 10^6^/kg of HCAR-19 cells for Phase 2 dosing (P2D) was established to maximize efficacy without significant toxicity, allowing for differential dosing based on host factors or prior disease burden without compromising safety or efficacy. This dose is currently being evaluated in the roll-on in a multi-centric Phase 2 registration trial, with ImmunoACT as the manufacturing site and co-sponsor.

## Supplementary information

Supplementary figure 1

Supplementary table 1

## Data Availability

The data supporting the findings of this study are available upon request from the corresponding author. To request access to the data, please contact Dr. Gaurav Narula at drgauravnarula@gmail.com.
