# Correction: Novel humanized CD19-CAR-T (Now talicabtagene autoleucel, Tali-cel™) cells in relapsed/ refractory pediatric B-acute lymphoblastic leukemia- an open-label single-arm phase-I/Ib study

**DOI:** 10.1038/s41408-025-01308-7

**Published:** 2025-05-20

**Authors:** Gaurav Narula, Swaminathan Keerthivasagam, Hasmukh Jain, Sachin Punatar, Akanksha Chichra, Chetan Dhamne, Prashant Tembhare, Papagudi Ganesan Subramanian, Nikhil Patkar, Minal Poojary, Anant Gokarn, Sumeet Mirgh, Nishant Jindal, Albeena Nisar, Deepali Pandit, Khushali Pandit, Alka Dwivedi, Atharva Karulkar, Ankesh Kumar Jaiswal, Aalia Khan, Shreshtha Shah, Afrin Rafiq, Moumita Basu, Juber Pendhari, Sweety Asija, Ambalika Chowdury, Ankit Banik, Nirmalya Roy Moulik, Shyam Srinivasan, Shilpushp Bhosle, Sumathi Hiregoudar, Shashank Ojha, Lingaraj Nayak, Jayshree Thorat, Bhausaheb Bagal, Manju Sengar, Navin Khattry, Shripad Banavali, Steven Highfill, Nirali N. Shah, Rahul Purwar

**Affiliations:** 1 Department of Pediatric Oncology, Tata Memorial Centre, Homi Bhabha National Institute, Mumbai, India; 2 Department of Medical Oncology, Jawaharlal Institute of Postgraduate Medical Education and Research, Puducherry, India; 3 Department of Medical Oncology, Tata Memorial Centre, Homi Bhabha National Institute, Mumbai, India; 4 Bone marrow transplant unit, Department of Medical Oncology, Tata Memorial Centre, Advanced Centre for Treatment, Research and Education in Cancer (ACTREC), Homi Bhabha National Institute, Mumbai, India; 5 Department of Hematopathology, Tata Memorial Centre, Advanced Centre for Treatment, Research and Education in Cancer (ACTREC), Homi Bhabha National Institute, Navi Mumbai, India; 6 Department of Transfusion Medicine, Tata Memorial Centre, Advanced Centre for Treatment, Research and Education in Cancer (ACTREC), Homi Bhabha National Institute, Navi Mumbai, India; 7 Scientific Officer (D), CAR-T and Cell Therapy Centre, ACTREC, Tata Memorial Centre, Mumbai, India; 8 CAR-T and Cell Therapy Centre, ACTREC, Tata Memorial Centre, Kharghar, India; 9 Department of Biosciences & Bioengineering, Indian Institute of Technology Bombay, Mumbai, India; 10 Pediatric Oncology Branch, Center for Cancer Research, National Cancer Institute, National Institutes of Health, Bethesda, MD USA; 11 Immunoadoptive Cell Therapy Private Limited (ImmunoACT), Mumbai, India; 12 Department of Critical Care and Anaesthesiology, Tata Memorial Center, Homi Bhabha National Institute, Mumbai, India; 13 Director Academics and Professor Medical Oncology, Tata Memorial Center, Homi Bhabha National Institute, Mumbai, India; 14 Center for Cellular Engineering, National Cancer Institute, National Institutes of Health, Bethesda, MD USA

**Keywords:** Acute lymphocytic leukaemia, Targeted therapies

Correction to: *Blood Cancer Journal* 10.1038/s41408-025-01279-9, published online 24 April 2025

In this article, the figure legend of Fig. 3 was given erroneously. Specifically, in the legend the colors representing CR and PR have been interchanged.

In the figure, the green bars correctly represent CR, and the blue bars correctly represent PR.

However, in the legend, the boxagainst CR has the blue color, and the box adjacent to PR has green color.

The correction needed is to change the colors of these boxes so that the box next to CR shows the green color and the box next to PR shows the blue color.
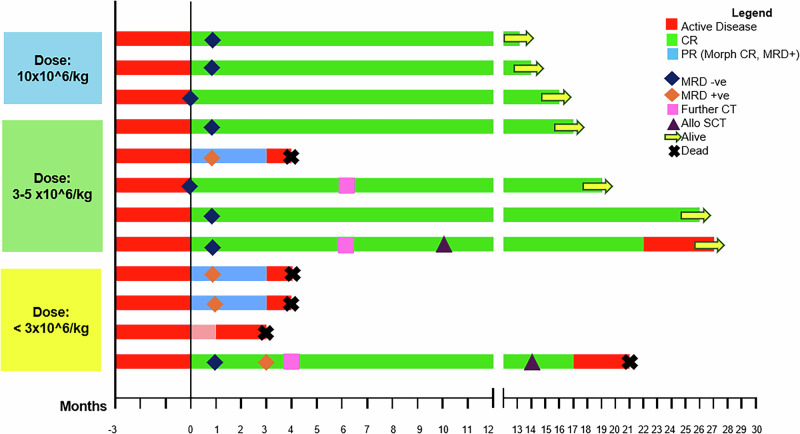


The original article has been updated.

