# Supplementary material for: Novel humanized CD19-CAR-T (Now talicabtagene autoleucel, Tali-cel™) cells in relapsed/ refractory pediatric B-acute lymphoblastic leukemia- an open-label single-arm phase-I/Ib study
Source: Blood Cancer J. 2025 Apr 24;15(1):75. doi: 10.1038/s41408-025-01279-9 (PMC12022059; doi:10.1038/s41408-025-01279-9)
Supplement: Supplementary file 1 — Supplementary figure 1 [file 41408_2025_1279_MOESM1_ESM.docx]

Supplementary Figure 1: Levels of other cytokines monitored at different time points for each patient
