# Supplementary material for: Novel humanized CD19-CAR-T (Now talicabtagene autoleucel, Tali-cel™) cells in relapsed/ refractory pediatric B-acute lymphoblastic leukemia- an open-label single-arm phase-I/Ib study
Source: Blood Cancer J. 2025 Apr 24;15(1):75. doi: 10.1038/s41408-025-01279-9 (PMC12022059; doi:10.1038/s41408-025-01279-9)
Supplement: Supplementary file 2 — Supplementary table 1 [file 41408_2025_1279_MOESM2_ESM.docx]

**Supplementary Table-1:** Clinical profile and course of a child with Burkitt lymphoma treated with HCAR-19

| **Variable** | **Details** |
| --- | --- |
| Clinical details | - 14 years old boy with Refractory Burkitt lymphoma- stage 4  - Progressive disease while treatment on LMB 96 regimen |
|  |  |
| Therapy prior to CAR-T infusion | - LMB 96 regimen - Prephase at progression (COP) |
| Total viable CAR-T cells infused/Kg | 4.9 million cells |
| Adverse events | Grade-2 CRS |
| Result | Clinically progressive disease during 3^rd^ week of HCAR-19 infusion |

(LMB-96: Lymphome Malins de Burkitt, COP- Cyclophosphamide- 300 mg/m^2^ on day 1, Vincristine- 1 mg/m^2^ on day 1, Prednisolone- 40 mg/m^2^ for 5 days, CRS- cytokine release syndrome)
